# Recent sylvatic yellow fever virus transmission in Brazil: the news from an old disease

**DOI:** 10.1186/s12985-019-1277-7

**Published:** 2020-01-23

**Authors:** Natalia Ingrid Oliveira Silva, Lívia Sacchetto, Izabela Maurício de Rezende, Giliane de Souza Trindade, Angelle Desiree LaBeaud, Benoit de Thoisy, Betânia Paiva Drumond

**Affiliations:** 1 Laboratório de Vírus, Departamento de Microbiologia, Instituto de Ciências Biológicas, Universidade Federal de Minas Gerais, Belo Horizonte, Minas Gerais Brazil; 2 Division of Infectious Disease, Department of Pediatrics, Stanford University School of Medicine, Stanford, California USA; 3 Laboratoire des Interactions Virus-Hôtes, Institut Pasteur de la Guyane, Cayenne, French Guiana

**Keywords:** Yellow fever virus, Yellow fever, Arbovirus, Flavivirus, Non-human primate, Epizootic, Outbreak, Epidemiology, Vector, Pathogenesis

## Abstract

Yellow fever (YF) is an acute viral disease, affecting humans and non-human primates (NHP), caused by the yellow fever virus (YFV). Despite the existence of a safe vaccine, YF continues to cause morbidity and mortality in thousands of people in Africa and South America. Since 2016, massive YF outbreaks have taken place in Brazil, reaching YF–free zones, causing thousands of deaths of humans and NHP. Here we reviewed the main epidemiological aspects, new clinical findings in humans, and issues regarding YFV infection in vectors and NHP in Brazil. The 2016–2019 YF epidemics have been considered the most significant outbreaks of the last 70 years in the country, and the number of human cases was 2.8 times higher than total cases in the previous 36 years. A new YFV lineage was associated with the recent outbreaks, with persistent circulation in Southeast Brazil until 2019. Due to the high number of infected patients, it was possible to evaluate severity and death predictors and new clinical features of YF. *Haemagogus janthinomys* and *Haemagogus leucocelaenus* were considered the primary vectors during the outbreaks, and no human case suggested the occurrence of the urban transmission cycle. YFV was detected in a variety of NHP specimens presenting viscerotropic disease, similar to that described experimentally. Further studies regarding NHP sensitivity to YFV, YF pathogenesis, and the duration of the immune response in NHP could contribute to YF surveillance, control, and future strategies for NHP conservation.

## Background

Yellow fever virus (YFV) causes yellow fever (YF), an acute disease affecting humans and non-human primates (NHP) in several South American and African countries [1, 2]. In humans, YF ranges from asymptomatic infection to non-specific symptomatic illness and fatal hemorrhagic fever [1, 2]. Despite the existence of a safe vaccine, massive YF outbreaks occurred recently in Angola, Democratic Republic of Congo [3], and Brazil [4–6]. Humans are considered incidental hosts for YFV. The virus is maintained by different cycles in tropical and subtropical regions of sub-Saharan Africa and South America via transmission among different mosquitoes and NHP [2, 7–9]. Most of the experimental studies with YFV were performed with Old World NHP, but little is known about YF and the pathogenesis in Neotropical NHP. Neotropical NHP are considered highly susceptible to YFV infection [10], which may be related to the recent introduction of the virus into the Americas [7]. Given the ongoing YF outbreaks in Brazil (2016–2019), we present the main aspects of sylvatic YF, including historical and recent outbreaks in the country.

## Main text

### The virus and transmission cycle

YFV is the prototype member of the genus *Flavivirus* (family Flaviviridae), with a single-strand positive-sense RNA genome of approximately 11 kb [11]. The genome has a 5′ end cap structure, and it is translated into a polyprotein precursor. The polyprotein is then cleaved by viral and cellular proteases into three structural proteins (capsid, envelope, and membrane proteins) and seven non-structural proteins (named NS1, NS2A, NS2B, NS3, NS4A, NS4B, and NS5) [11].

Until now, one YFV serotype and seven genotypes have been described in Africa and South America. In Africa, five genotypes are described, named West Africa I, West Africa II, East Africa, East/Central Africa, and Angola [12, 13]. In Africa, there are three transmission cycles: (*i*) the sylvatic cycle is reported in the rainforest, involving NHP and sylvatic *Aedes africanus*, (*ii*) the intermediate cycle in the forest-savanna ecotone has peridomestic anthropophilic *Aedes spp.* (such as *A. furcifer*, *A. taylori*, *A. luteocephalus*, and *A. simpsoni*) as vectors, and (*iii*) the urban cycle has *Aedes aegypti* and *Aedes bromeliae* as vectors [12, 13]. In South America, YFV is endemic in the Amazon Basin (Brazil, Peru, Bolivia, Colombia, Ecuador, Venezuela, French Guiana, Suriname, and Guyana) [1, 14] and the sylvatic cycle involves various species of NHP and mosquitoes primarily belonging to the genera *Haemagogus and Sabethes* [15]. YFV South American I and II genotypes are derived from the West African genotype [16, 17]. South American I is the predominant YFV genotype in Brazil, possessing five distinct lineages 1A-1E [17, 18]. Until the middle of the 1990s, the old lineages (1A, 1B, and 1C) co-circulated in South America but were then replaced by the modern ones, 1D and 1E [8, 17–19]. Lineage 1E is responsible for the recent YF outbreaks in Brazil (2016 to 2019), and it was probably originated from YF endemic areas in North Brazil [20, 21]. Genomic analyses of YFV causing the recent outbreaks revealed unique mutations leading to nine amino acid substitutions in the deduced polyprotein (eight substitutions in highly conserved positions of non-structural proteins 3 and 5, which form the replication complex of YFV). Those amino acid substitutions have not been previously described for YFV, and the impacts of those changes in viral fitness should be investigated [19, 22].

### Recent clinical findings in yellow fever patients

YF has been described as a viscerotropic disease in humans, with viral replication playing a crucial role in pathogenesis [23]. The viscerotropic YF has been divided into three periods: (i) infection, characterized by viremia and occurrence of flu-like symptoms; (ii) remission, when seroconversion is observed while fever and symptoms bounce back or disappear; and (iii) intoxication, which affects 15–25% of symptomatic patients. During the intoxication period, symptoms reappear, including hemorrhagic fever, multi-organ dysfunction, jaundice, oliguria, anuria, renal failure, and cardiovascular instability [1, 23]. Among the severe cases, global mortality varies from 5 to 10%, but 40% of lethality has already been described in Brazil [2].

During the recent epidemics in Brazil, the most common signs and symptoms observed in humans were fever, headache, vomiting, jaundice, chills, nausea, abdominal pain, myalgia, arthralgia, rash, diarrhea, bleeding or hemorrhagic signs [24–27]. Recently, in severe YF cases, a critical metabolic acidosis leading to the need for hemodialysis [25], increased levels of serum lipase [25, 28] and a high prevalence of pancreatitis were observed [25]. These studies highlighted the importance of pancreatitis in the evolution of YF and the need for further studies addressing this issue [25, 28].

Other studies have demonstrated different outcomes and patterns regarding YF infection. Although YF is mainly viscerotropic in humans, Marinho and colleagues (2019) described a case of a 3-year-old girl with severe manifestations in the central nervous system. The patient had mildly elevated amino transaminases but no classical YF signs or symptoms. After six days of hospitalization, the child died, and postmortem analysis showed wild-type YFV RNA in the cerebrospinal fluid [29]. The persistence of the wild-type YFV genome has been demonstrated in serum [28] and in urine [28, 30], until 28 and 47 days after the onset of symptoms, respectively. These findings indicate that YFV might persist in the host for more extended periods than previously thought. During the follow-up of two YF patients, increased levels of serum alanine transaminase (ALT) and serum aspartate transaminase (AST) (ALT > 1000 IU/L and AST > 301 IU/L) were observed two months after the onset of YF. The serum transaminases persisted elevated for up to six months [31], indicating the need for further studies on the convalescence phase of YF.

Studies have also been conducted to investigate the predictors of severity and death in different groups of YF patients. Age and elevated levels of AST, ALT, and creatinine have been independently associated with mortality in YF patients [26], while higher values of serum lipase and lower values of factor V were related to severe cases [28]. Kallas and colleagues (2019) analyzed data from 76 YF patients and observed that age, neutrophil count, AST, and viral load were independently related with death [27]. Clinical and laboratory indicators could support the management of patients and the establishment of prognosis. However, one should keep in mind that YF evolves rapidly, and clinical characteristics may vary according to different factors related to the host and the phase of infection.

### Yellow fever surveillance and control

Since all South American NHP are highly susceptible to YFV infection, and epizootics often precede human cases, NHP can be used as sentinels during surveillance programs [8]. In 1999, the Brazilian Ministry of Health (MOH) launched the Epizootics Surveillance Program aiming at the investigation of NHP epizootics and entomological surveillance [32]*.* After the detection of epizootics causing the death of NHP in a region, a survey of the vaccination history of residents near the site, an active search for suspected human cases, and biological sample collection for laboratory investigation are conducted. For YF diagnosis in NHP, organ fragments, especially the liver, but also spleen, kidney, heart, lungs, and brain are collected and forwarded to one of the Reference Laboratories linked to the MOH. These samples are used for investigation by immunohistochemistry, histopathology, viral isolation, or by molecular tests [33]. Immunohistochemistry and histopathology analyses allow the detection of YFV antigens and the identification of histological alterations observed in YF, respectively. Although there is scarce information regarding the infection of monkeys by other flaviviruses, some studies have detected Zika virus RNA in NHP, during the latest YFV outbreaks in Brazil [34, 35]. Faced with this, molecular techniques such as RT-qPCR have become the most reliable and specific tool to diagnose and confirm YFV infection.

YF laboratory diagnosis is performed through virologic or serological methods using human samples, by the Reference Laboratories linked to the MOH. For molecular detection of YFV RNA by RT-qPCR, sera collected from the first up to the 10th day after the onset of symptoms is recommended, and the positive result confirms YFV infection. Serological tests, especially MAC-ELISA to detect IgM antibodies, are recommended to be used after seroconversion, from the sixth day after the onset of symptoms. Due to the high probability of cross-reactivity with other flaviviruses, which are widespread throughout the same regions of YFV occurrence, the detection of IgM anti-YFV is only presumptive of recent infection [36, 37].

Since YF is a disease of immediate compulsory notification, the suspected human cases and NHP epizootics should be reported within 24 h of initial suspicion [32, 37]*.* Surveillance and precise diagnosis of YF are essential since they may support control measures such as vaccination [38]*.* YF vaccination recommendation was expanded in Brazil and now includes all states from North, South, Southeast, and Midwest regions and parts of the Northeast region (Maranhão, Bahia, and some municipalities in Piauí, Alagoas, and Sergipe) [6]. A single dose of the YF vaccine is recommended for lifelong protection [39, 40], however, this is still an issue of debate. Studies have demonstrated a significant decreasing or even the complete absence of neutralizing antibody titers, effector memory CD4^+^ and CD8^+^ T-cells and classical memory B-cells ten years after primary YF vaccination [41–43].

Regardless of the relative safety of the vaccine strains, some adverse events following YF vaccination have been described [38, 44]. In Brazil, from 2007 to 2012, the occurrence of adverse events was estimated as 0.42 events per 100,000 inhabitants [45]. In recent epidemics in Minas Gerais state from 2017 to 2018, more than 7.1 million YFV-17DD vaccine doses were given, and only one case of vaccination-associated disease was confirmed to date [46].

Much is debated on the vaccination of NHP against YF. The immunization of NHP could reduce virus circulation significantly in risk areas and, consequently, the chances of infection in humans. Besides, this strategy could help the preservation and protection of monkey populations. On the other hand, some points need to be taken into consideration. There are not enough studies regarding the effect of the vaccine in Neotropical NHP and, given the differences in susceptibility to YFV, the vaccine dose would have to be adjusted for each species. There are also logistics and cost aspects related to the capture of the animals to be considered [47]. Finally, epizootics surveillance programs could fail in early detection of YFV circulation, due to the loss of naïve sentinel NHP.

### Sylvatic yellow fever in Brazil

During the 18th and 19th centuries, YFV caused devastating urban outbreaks in the Americas, and it was considered one of the most dangerous diseases of that period [12]. At the beginning of the twentieth century, campaigns focused on eradicating the *vector A. aegypti* started in several places in South America [48], leading to the eradication of the YF urban cycle in many countries, including Brazil. The last urban YF epidemic in Brazil was reported in 1929 in Rio de Janeiro state, and the ultimate YF urban case was documented in 1942, in Acre state [49].

The sylvatic cycle of YF in Brazil was observed in 1889, by Adolpho Lutz, and in 1899, by Emilio Ribas, when YF human cases occurred in *A. aegypti*-free areas, in São Paulo state [50]. However, only in 1932, the occurrence of the sylvatic cycle of YF was confirmed in Espírito Santo state [51]. In the following years (1933, 1934, 1935, 1936, 1938, 1939, 1944, 1950, 1957, and 1964), several sylvatic YF outbreaks were reported all over Brazil, in North (Amazonas and Pará states), Northeast (Bahia state), Midwest (Mato Grosso and Goiás states), Southeast (Minas Gerais, São Paulo, Rio de Janeiro, and Espírito Santo states), and South (Paraná, Santa Catarina, and Rio Grande do Sul states) regions. From 1932 to 1967, a total of 1672 human cases linked to sylvatic YF were confirmed [50]. Later, YF outbreaks were mostly reported in the Amazon Basin (North and Midwest regions), and sporadic cases occurred in Minas Gerais state [2, 52]. After 1999, the epidemiological scenario of sylvatic YF changed, and the majority of human cases occurred outside the Amazon Basin, in the Midwest, Southeast, and South regions of Brazil [2, 52]. From 1980 to mid-2015, 792 sylvatic YF human cases and 421 deaths took place in the country (data received from SINAN, June 2019) .

In 2016, a sylvatic YF outbreak started in Minas Gerais state, and then it spread to São Paulo, Espírito Santo, Rio de Janeiro, and Bahia states in 2017 [4, 5]. The YFV lineage causing this outbreak was estimated to exist for at least two years before the outbreak and likely reached the Southeast region by passing through the Midwest region of Brazil [53, 54]. YFV-positive NHP were detected in Minas Gerais state throughout 2017 until mid-2018 [55, 56]. These findings raised the possibility of viral persistence in the Southeast region, which was later confirmed [54, 57]. YFV cases occurred until 2018 [53, 54] in Minas Gerais, Rio de Janeiro, Espírito Santo, and São Paulo states [5]. In 2019, YF outbreaks were reported in São Paulo, and the virus spread to Santa Catarina and Paraná states [6].

**Fig. 1:**
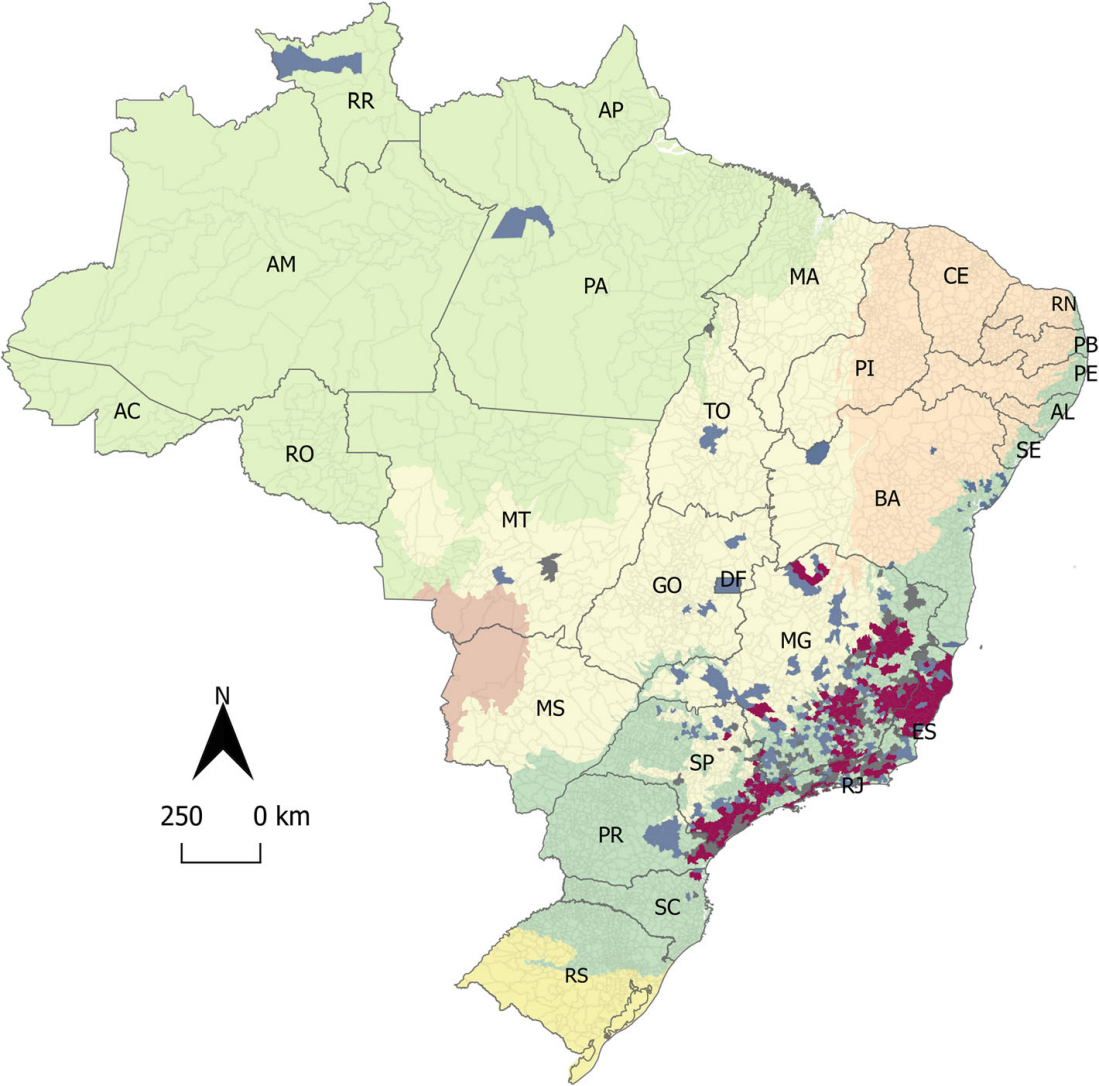
Sylvatic yellow fever (YF) human cases and epizootics occurrence in Brazil, from 2016 to 2019. The map presents the Amazon (in light green), Caatinga (in light orange), Cerrado (in light yellow), Atlantic Forest (in dark green), Pampa (in dark yellow), and Pantanal (in light brown) biomes in Brazil. Municipalities are colored according to YF cases in non-human primate (NHP) (in blue), in humans (in grey), and both in humans and NHP (in red). Brazilian states abbreviations are as follows: North: AC (Acre), AP (Amapá), AM (Amazonas), PA (Pará), RO (Rondônia), RR (Roraima), and TO (Tocantins); Northeast: AL (Alagoas), BA (Bahia), CE (Ceará), MA (Maranhão), PB (Paraíba), PE (Pernambuco), PI (Piauí), RN (Rio Grande do Norte), and SE (Sergipe); Midwest: MT (Mato Grosso), MS (Mato Grosso do Sul), GO (Goiás), and DF (Federal District/Brasília); Southeast: MG (Minas Gerais), SP (São Paulo), RJ (Rio de Janeiro), and ES (Espírito Santo); South: RS (Rio Grande do Sul), PR (Paraná), and SC (Santa Catarina). The map was created using QGIS v.2.18.16 [59]. The numbers of YF cases were obtained from Sistema de Informação de Agravos de Notificação (SINAN) and official bulletins from the Brazilian Ministry of Health Brazil and State Health Departments [60–73]. YF: yellow fever. H: humans. NHP: non-human primates

The 2016–2019 YF outbreaks in Brazil have been considered the most significant ones of the last 70 years. From December 2016 until June 2019, more than 15,000 NHP epizootics were reported in Brazil [4–6], in sylvatic, rural, and urban areas, as reported by local media [58]. Laboratory tests or epidemiological criteria confirmed at least 1567 epizootic events caused by YF, in 455 municipalities, in Southeast (90.9%), South, Northeast, Midwest, and North regions (Fig. 1) [55, 60–73].

**Fig. 2:**
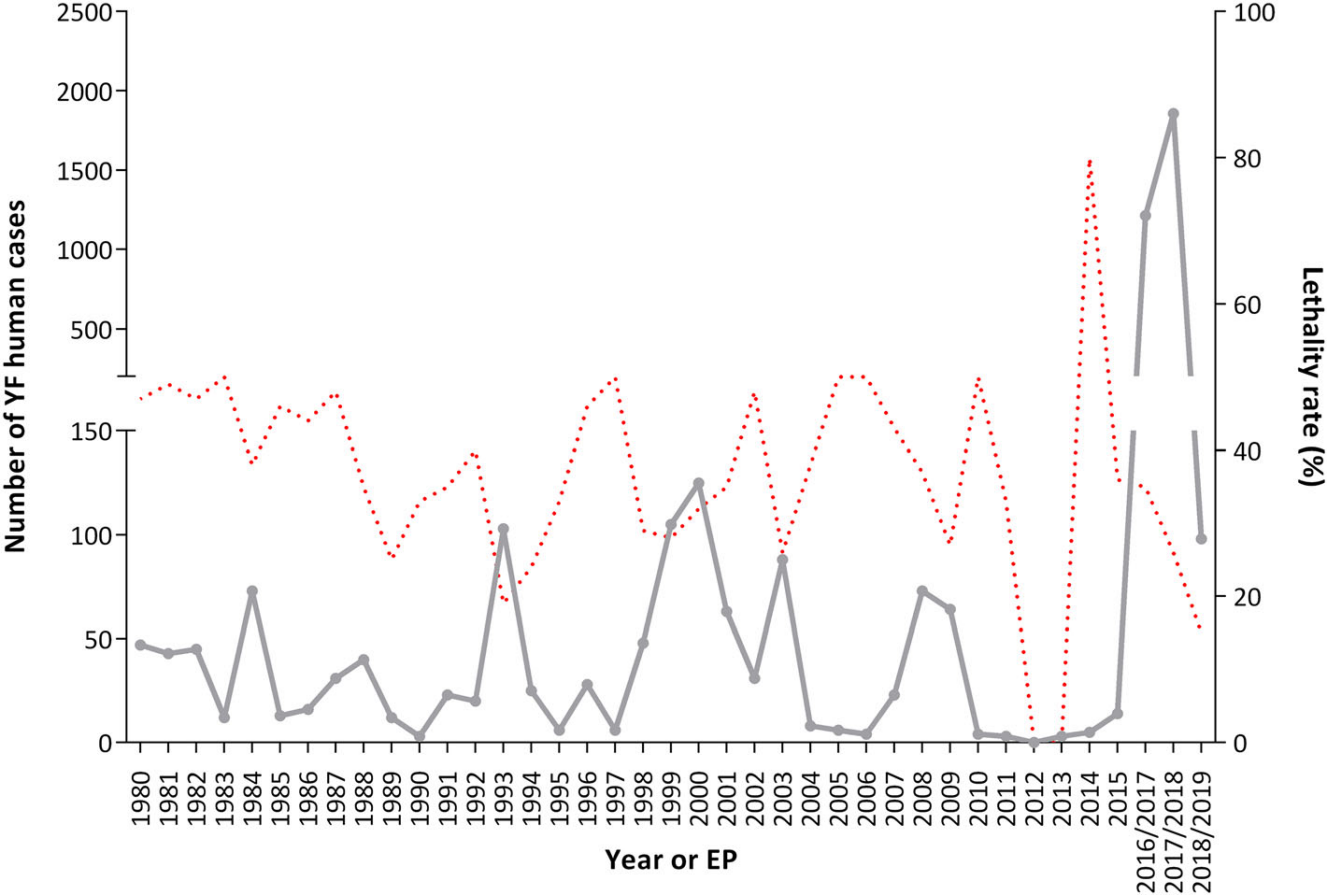
Historical series of sylvatic yellow fever human cases in Brazil, from 1980 to June 2019. The numbers of cases (grey line) and the lethality rate (red dotted line) are showed by year (1980–2015) or by epidemiological periods (EP) (July to June of 2016/2017, 2017/2018, and 2018/2019). The numbers of YF cases were obtained from Sistema de Informação de Agravos de Notificação (SINAN) and official bulletins from the Brazilian Ministry of Health Brazil and State Health Departments [60–73]

In Brazil, most of human YF cases are described in males aged 14 to 35, who are more exposed to YFV by occupational or ecotourism activities in rural and sylvatic endemic regions. However, since the end of the 1990s, the numbers of YF cases among women and younger people have been increasing [2]. At least 2251 human cases and 772 deaths were confirmed in Brazil, from December 2016 to June 2019 [4–6]. These numbers demonstrate an increase of 2.82 times the total of YF human cases and 1.57 times the sum of YF human deaths, compared to the previous 36 years (from 1980 to 2015) (Fig. 2). Human cases were confirmed in 388 municipalities in the Southeast (96%), South, Midwest, and North regions [60–73] (Fig. 1). These data illustrate the magnitude of the recent YF epidemics in Brazil, reaching extensive YF-free areas.

### Yellow fever virus vectors in Brazil

Unlike NHP and humans, which succumb to the disease or heal by developing long-term immunity, mosquitoes are considered reservoirs of YFV since they remain infected and can transmit the virus throughout life [2, 74]. In Brazil, species within the genera *Haemagogus* (*H. janthinomys*, *H. albomaculatus*, *H. leucocelaenus*, *H. capricornii*, and *H. spegazzinii*) and *Sabethes* (*S. chloropterus*, *S. soperi*, *S. cyaneus*, *S. glaucodaemon*, and *S. albiprivus*) are the primary sylvatic YFV vectors [2, 33, 75]. YFV-naturally infected specimens of *Aedes scapularis*, *Aedes taeniorhynchus*, *Aedes serratus*, and *Psorophora ferox* have been reported in Brazil, however, these mosquitoes are not considered primary YFV vectors [75–79].

*H. janthinomys* and *H. leucocelaenus* have a wide distribution in the South American continent and are considered the main YFV vectors in the Americas and South/Southeast Brazil, respectively [80, 81]*.* Females of *Haemagogus* spp. are hematophagous and exhibit primatophilic daily activity. They have vertically stratified spatial distribution and are mostly found in the forest canopy (acrodendrophilic behavior) [82, 83]. Female mosquitoes prefer oviposition in phytotelmata, such as tree hollows, bamboo internodes, bromeliads and leaf axis [84]. Later on, the immature stages (eggs, larvae, and pupa) develop in those phytotelmata. *Haemagogus* females seek and bite their hosts mostly in the afternoon or during the warmest hours of the day [2, 76, 80, 85].

*Sabethes* spp. share some ecological characteristics with *Haemagogus* spp., such as the daily activity, primatophilia, acrodendrophilia, and preferential oviposition in tree hollows. While some species are widespread in Central and South America (*Sabethes chloropterus* and *Sabethes cyaneus*), others seem to be restricted to South America (*Sabethes glaucodaemon*, *Sabethes soperi*, and *Sabethes albiprivus*) [86]*.* Within the genera, *S. chloropterus* is limited to forest areas and considered the primary YFV sylvatic vector. Their eggs can persist through the dry season, which is probably a key factor in YFV maintenance through unfavorable dry periods [86].

Sylvatic YFV vectors are abundant in the treetops, with daytime activity peaking in the hottest hours of the day, corresponding to the resting period of the NHP in the canopy [87], favoring the hematophagy in NHP. *H. leucocelaenus* and *H. janthinomys* are primatophilic species, but occasionally they can be found at ground level in areas surrounding [88] or distant from the forests, including indoors [89], since they can can travel for long distances, as 5.7 km and 11.5 km, respectively [90]. *H. leucocelaenus* can adapt to human-modified environments, as periurban [83, 86] and urban areas [91]. YFV-infected specimens of *H. janthynomis* have already been sampled in regions near or contiguous to urban neighborhoods [75, 92]. In that way, these mosquito species could play a significant role in viral maintenance in forest settings and in viral dissemination towards peridomestic and urban environments.

Given the ecological plasticity and the ubiquitous environmental distribution of *A. albopictus* (in urban, rural, and forest settings in tropical countries, including Brazil), this mosquito could be a bridge vector between sylvatic and urban environments [89, 93]. Experimental studies have demonstrated that *A. albopictus* can transmit YFV [77, 94–96], and it was found naturally infected with the virus, in a rural setting of Minas Gerais state, in 2018 [97]. *Aedes aegypti* is widespread in all the tropical and subtropical regions. This mosquito has anthropophilic and endophilic habits and is the urban vector of YFV [81]. Despite the sensitivity to YFV infection and the high infestation indices of *A. aegypti* and *A. albopictus* in urban and periurban areas in Brazil [75, 77, 98, 99], there is no evidence of YFV transmission by *A. aegypti*, since the eradication of urban YF cycle in the country [48, 75].

### Non-human primates in Brazil

Neotropical NHP are divided into five families (Pitheciidae, Aotidae, Atelidae, Cebidae, and Callitrichidae) [100, 101]. The most considerable diversity of NHP is observed in Brazil, with a total of 130 species mostly distributed in the Amazon, followed by Atlantic Forest, Caatinga, Cerrado, Pantanal, and Pampa biomes (https://www.taxeus.com.br/lista/3035).

The family Pitheciidae comprises NHP without prehensile tails which use quadrupedal locomotion, and most of the species are herbivorous. The species within the genera *Pithecia*, *Cacajao,* and *Chiropotes* are distributed in the Amazon region. However, *Callicebus,* the species-richest genus of Neotropical NHP, has a wide occurrence in the Brazilian territory in areas covered by the Amazon, Atlantic Forest, Caatinga, and Pantanal biomes [100, 102]. *Callicebus* specimens are much smaller than the other members of the family (0.8–1.5 kg) and differ in social organization, living in small family groups with a single monogamous adult pair. They mainly eat fruits, but also leaves, flowers, insects, bird eggs, and small vertebrates [100, 102].

The family Aotidae has one genus, *Aotus,* popularly called owl monkeys. Six out of eight *Aotus* species are distributed in the Brazilian Amazon. They are small (0.8 kg to 1.0 kg) and have nocturnal habits [102]. The owl monkeys live in small groups of 4–6 individuals and feed mainly on leaves, fruits, and invertebrates [100].

The family Atelidae comprises the largest Neotropical NHP (5 to 15 kg). They have a prehensile tail, are excellent brachiators, and feed mostly on flowers and fruits. The spider monkeys (*Ateles*) and woolly monkeys (*Lagothrix*) are distributed in the Amazon, while the muriquis (*Brachyteles)* are observed in the Atlantic Forest. The howler monkeys (*Alouatta*), recognized by their loud and characteristic vocalization, have slow locomotion and live in small tight groups. Ten species of *Alouatta* are widespread in the Amazon Basin, Atlantic Forest, Cerrado, Caatinga, Pantanal, and Pampa biomes [101, 102].

The family Cebidae comprises medium-sized animals, which live in large social groups. These NHP are mostly are frugivorous and insectivorous. *Sapajus*, *Cebus*, and *Saimiri* are found in the Amazon, although *Sapajus* specimens have a broad distribution in the Atlantic Forest, Cerrado, Pantanal, and Caatinga biomes [101, 102].

The family Callitrichidae comprises small animals (120 g to 600 g) that feed on fruits, insects, and plant exudates. The genera *Callimico, Callibella, Cebuella,* and *Saguinus* are distributed in the Amazon, while *Leontopithecus* is present in the Amazon, Pantanal, and Cerrado biomes. The *Callithrix* genus, usually called marmoset, is distributed in the Atlantic Forest, Cerrado, and Caatinga biomes [101, 102].

As seen above, a great variety of arboreal NHP is found in different geographical areas where the presence of sylvatic vectors is registered. The urbanization and the expansion of agriculture practices have led to extreme habitat degradation, causing the fragmentation NHP populations in Brazil [103]. Although NHP mostly exist in sylvatic environments, some Neotropical NHP adapt and survive in extremely degraded habitats [104, 105]*.* Neotropical NHP are potential hosts for YFV due to their ecological and behavioral patterns and their susceptibility to the infection. A variety of factors could alter or lower the exposure rates of NHP to hematophagous insects. For example, the small body and group sizes of some monkeys lead to lower rates of malaria in Amazonian NHP [106]. Some NHP species exhibiting less mobility, with territorial and quite habits in the tree canopy, as *Alouatta* spp., have higher chances of being bitten and infected [103, 107]. Social and diet behavior patterns could be modified in disturbed habitats, as already shown for the red howler monkey [108]. Those behavior changes could, in turn, alter the attractiveness in favor of hematophagous mosquitoes and, consequently, the transmission of viruses.

### Yellow fever in non-human primates

Old World NHP are sensitive to YFV and develop enough viremia to reinfect mosquitoes but rarely they show clinical signs or die. In Africa, the primary YFV amplifier hosts are *Cercopithecus* spp. (with viremia lasting for three to four days) [86] and *Colobus* spp. (with viremia detected up to nine days) [109]. Serological studies indicate that *Cercocebus* spp. (mangabeys) and *Papio* spp. (baboons) [110, 111], and possibly *Galago senegalensis* could be infected and contribute to YFV transmission [7]. Serological and clinical findings related to YFV infection are described in many Neotropical NHP [112–114]. All Neotropical NHP genera are considered susceptible to YFV infection with differing degrees of sensitivity [115, 116].

Early reports in Brazil showed antibodies against YFV in specimens of *Cebus, Callithrix,* and *Leontopithecus*, in Mato Grosso, Bahia, and Minas Gerais states [117–119]. An extensive immunological survey detected humoral immunity against YFV in *Alouatta*, *Ateles*, *Aotus*, *Callicebus*, *Callithrix*, *Cebus*, *Brachyteles*, *Leontopithecus*, *Pithecia*, and *Saimiri* in Brazil [120]. A recent study [121], reported the decline (10 to 26%) of two muriqui populations (*Brachyteles hypoxanthus*), from October 2016 to April 2017, in areas with YF outbreaks [55], and likely YF was the cause of deaths [121].

*Alouatta* is described as the most susceptible genus to YFV infection, developing clinical illness and fatal disease [114, 122, 123], even when infected with low virus inoculum [2]. However, YFV antibodies have been detected in some specimens of *Alouatta*, showing that some animals survive [2, 120, 124, 125]. In South America, it is common to hear howling monkeys (*Alouatta* spp.) in the forests, a territorial habit, and their sudden silence is considered a signal for the circulation of YFV [33, 89]. High numbers of NHP deaths, especially of *Alouatta* spp., have been associated with YF outbreaks in Argentina [115, 126], Costa Rica [127], Panama [128], Venezuela [129, 130], and Brazil [9, 20, 131, 132].

Specimens of *Callithrix* are also involved in the sylvatic cycle of YFV in Brazil. Experimental studies demonstrated the susceptibility of *Callithrix albicolis*, *Callithrix penicillata,* and hybrids marmosets to YFV [113]. Wild specimens of *C. penicillata* presenting histopathological findings suggestive of YFV infection, and marmosets showing anti-YFV antibodies have been reported in many parts of Brazil [120, 133]. In general, species within the genera *Saimiri*, *Saguinus*, *Aotus*, *Ateles*, *Cebus,* and *Sapajus* have been considered less susceptible to YFV, although *Saimiri, Saguinus* and *Sapajus* specimens may develop the disease, with fatal outcomes [9, 20, 89, 114, 134, 135].

Since the beginning of the most recent YF outbreak in Brazil (2016–2018), several epizootics have been reported by the MOH and other studies, affecting mostly marmosets and howler monkeys [20–22, 33, 53, 54, 136, 137]. YFV-infected specimens of *Sapajus libidinosus, Alouatta caraya,* and *Alouatta clamitans* have been described (2015–2017) in Goiás and Espírito Santo states [53]. From 2017 on, genomic and epidemiological studies described YFV infection in NHP in the Southeast region of Brazil. YFV-infected specimens of *Alouatta* spp., *Alouatta clamitans, Callithrix* spp., *Callithrix jacchus, Callithrix penicillata*, *Callicebus* spp., and *Callicebus personatus* were observed in Minas Gerais, Espírito Santo, and Rio de Janeiro states [21, 22, 54, 57, 136–138]. During an investigation of YF epizootics in São Paulo from 2016 to 2017, Cunha and colleagues (2019) confirmed the infection in specimens of *Alouatta, Callithrix,* and *Sapajus*. Based on molecular and immunohistochemistry analyses, the authors suggested that some species of *Callithrix* may have different sensitivity to YFV when compared to *Alouatta* spp. and *Sapajus* spp. [20].

Although all Neotropical NHP have been considered sensitive to YFV, most of the records and studies are related to *Alouatta* and *Callithrix*. From 2221 NHP deaths, caused by YF, from 1996 until June 2016, in Brazil, the most affected NHP were *Alouatta* sp. (85.0%) followed by *Callithrix* sp. (8.3%), *Sapajus* sp. (0.8%), *Cebus* sp. (0.22%), and *Saimiri* sp. (0.05%). From July 2016 until April 2019, at least 3569 NHP deaths were caused by YF. From these total, most of the identified specimens were *Alouatta* sp. (31.7%) and *Callithrix* sp. (17.1%), followed by *Sapajus* sp. (1.1%) and *Cebus* sp. (1.0%). For the first time, SINAN registered cases of YF in *Callicebus* sp. (0.45%), *Aotus* sp. (0.03%), and *Ateles* sp. (0.03%). Also, the first case of YF was registered in *Leontopithecus rosalia* (golden lion tamarin), an endangered species native of Atlantic Forest (data received from SINAN, June 2019).

Some studies described NHP genera with different degrees of sensitivity to YFV, based on natural infection or few experimental assays. However, the susceptibility of NHP to YF is sometimes estimated using data of animals which died or survived after natural infection. It is also important to note that NHP species have a different distribution throughout Brazil, and their genetic background could influence their susceptibility to YFV. One could expect that NHP from the Amazon Basin, a YF endemic region, could be more resistant to YFV compared to NHP from YFV-free areas. Besides other ecological factors unique to each NHP genus/species, as geographic distribution and behavioral patterns could favor infection.

### Pathogenesis of yellow fever in non-human primates

Most of the knowledge about the pathogenesis of YF in humans and NHP results from experimentally infected rhesus monkeys (*Macaca mulatta*), *Alouatta* spp., and *Saimiri* spp. [114, 139]. The first successful infection of a rhesus monkey with YFV was demonstrated in 1928, by Stokes and colleagues (1928). The authors described the main pathological changes related to acidophilic necrosis of the liver parenchyma affecting predominantly the mid-zonal region of the lobule [140]. Similar findings were later reported in rhesus monkey three days post-inoculation with a high infectious dose (4565–400,000 times of the 50% lethal dose in a mice model). The progress of the disease was marked by an increase in necrotic liver cells, accompanied by an increase in animal temperature. Necropsies demonstrated infiltration of the portal tract and hepatic veins of the liver with inflammatory cells [141].

In general, NHP exhibit the viscerotropic disease, and YFV can be found in the liver, kidneys, bone marrow, spleen, and lymph nodes [142]. In rhesus monkeys, the viremia peaks from three to seven days post-infection (with estimated viral titers of 25 TCID_50_ to 5 × 10^4^ TCID_50_), coinciding with the observation of severe disease [142]. The infection and lesion of hepatocytes have been described as a relatively late event. Eosinophilic degeneration of hepatocytes presenting condensed nuclear chromatin (Councilman bodies), precedes (24-48 h) death in experimentally infected monkeys [1, 143]. Clinical observations of experimentally infected rhesus monkeys showed necrosis of germinal centers of the spleen, lymph nodes, tonsils, Peyer’s patches, and renal failure. Hypoxia, hypotension, and circulatory shock and multi-organ failure are also observed. A systemic inflammatory syndrome (cytokine storm) might contribute to the lethality of YF, but further studies are needed to better understand the role of immunopathological mechanisms during YF [1, 13, 143]. YFV-infected NHP may present fever, jaundice, dehydration, anorexia, oral and intestinal bleeding, liver injury with hemorrhagic and necrosis foci, and kidney failure [33, 137, 142].

There are few descriptions of YF histopathological lesions in YFV-naturally infected NHP, especially regarding Neotropical NHP. Leal and colleagues (2016) described liver necrosis in the midzone associated with apoptosis of hepatocytes as the most frequently histopathologic change in naturally infected howler monkeys (*Alouatta* spp). They also observed steatosis, liver hemorrhage, inflammatory mononuclear cell infiltration of the liver, renal acute tubular necrosis, and interstitial nephritis [139].

Recent YF outbreaks in Brazil opened an opportunity to study naturally infected NHP. Cunha and colleagues (2019) performed immunohistochemistry analyses and observed brown granular cytoplasmic hepatocytes in YFV-positive specimens of *Alouatta* spp. (*n* = 22), *Sapajus* spp. (*n* = 7), but not in five specimens of YFV-positive *Callithrix* [20]. Two carcasses of female *Callicebus personatus* displayed petechiae in the gastric mucosa, blood clots in the stomach, hemorrhagic and friable spleen, macroscopic changes with yellow areas in the hepatic parenchyma, plus edema in lungs and eyelid, and hyperemia of the eyelid. YFV RNA was detected in the lungs, liver, kidneys, bladder, stomach, and intestine, suggesting that the pathological picture was probably caused by YFV [137]. Fernandes and colleagues (2017) performed histologic analyses of NHP carcasses (two specimens of *Alouatta* sp and 22 non-identified specimens). They observed massive liver necrosis with Councilman bodies, splenic lymphoid depletion, follicular necrosis, acute renal tubular necrosis, and multisystemic hemorrhage [138]. One animal from this study presented atypical signs, such as massive steatosis with rare midzonal to random single-cell necrosis and multifocal micro abscesses in the liver, probably associated with an acute intestinal bacterial infection or septicemia. The same animal presented endothelial necrosis and hepatic regeneration signs, as suggested by oval cell hyperplasia [138]. During the last YF outbreak in Brazil, natural infection of Neotropical NHP resembled experimental infection, but new findings were reported. The comprehensive understanding of YF pathogenesis in Neotropical NHP could bear further studies using alternative animal models, bringing light in important aspects of YF pathogenesis and treatment.

## Conclusions

The changes in the epidemiological pattern of sylvatic YF since the 2000s and unprecedented magnitude of the latest YF outbreak in Brazil are an alert. A new YFV lineage was associated with the 2016–2019 outbreaks. However, whether the mutations observed in this new lineage contribute to viral fitness in vectors or hosts, and possibly to the dynamics of YF is still a matter of investigation. Genomic surveillance and evolutionary studies demonstrated the sustained circulation of YFV before December 2016 until 2019, reaching extensive YF-free areas with a significant number of naïve hosts (human and NHP) and competent vectors.

Although YF is an old disease, much is still unknown about the pathogenesis, clinical aspects of the disease, and patient management. During recent outbreaks in Brazil, the study of a high number of YF patients, who were admitted at high-quality hospitals, brought an opportunity to elucidate some clinical features of the disease, but many gaps are still open, and further studies are needed.

The recent YF outbreaks allowed the investigation of a variety of NHP outside the Amazon Basin. Following the pattern previously observed, most YFV-infected specimens belonged to *Alouatta* spp. and *Callithrix* spp., and for the first time YF was described in *Leontopithecus rosalia.* NHP are amplifier hosts for YFV but are not viral reservoirs since they die or develop cellular and humoral immunity against the virus. However, studies to better understand the duration of protective immunity against YFV in NHP are required.

Further investigation of viremia in Neotropical NHP could contribute to the assessment of sensitivity to YFV and the potential for vector infection. This information is critical for YF surveillance, identification of YF risk-areas, and the establishment of YF control measures and future strategies for NHP conservation. Despite the imminent risk for YF re-urbanization, given YFV-infected NHP inside urban metropolitan areas of Brazil, no human case was epidemiologically linked to the YF urban transmission cycle. Further studies to understand the role of NHP in YFV circulation in different environments are needed, as well as a greater understanding of the impact of YF in NHP populations.

## Data Availability

The data that support the findings of this study are available from SINAN, but restrictions apply to the availability of these data, which were used under license for the current study, and so are not publicly available. Data are, however, available from the authors upon reasonable request and with permission of the Serviço de Informação ao Cidadão and SINAN/Brazilian Ministry of Health.

## Abbreviations

ALT: Alanine aminotransferase
AST: Aspartate aminotransferase
ICU: Intensive care unit
LACEN: Reference Laboratories/Brazilian Ministry of Health
MAC-ELISA: Immunoglobulin M antibody caption – Enzyme-linked immunosorbent assay
MOH: Brazilian Ministry of Health
NHP: Non-human primate
NS: Non-structural protein
RT-qPCR: Reverse transcription real-time Polymerase chain reaction
SINAN: Information System on Diseases of Compulsory Declaration/Brazilian Ministry of Health
TCID_50_: Median tissue culture infectious dose
YF: Yellow fever
YFV: Yellow fever virus

## References

[1] Monath TP (2001). Yellow fever: an update. Lancet Infect Dis.

[2] Vasconcelos PFC (2003). Yellow fever. Rev Soc Bras Med Trop.

[3] Maguire HCF, Heymann DL. Yellow fever in Africa. BMJ. 2016:i3764. 10.1136/bmj.i3764 27422309

[4] MS-BR. Centro de Operações de emergências em Saúde Pública sobre Febre Amarela – N^o^ 43/2017 [Internet]. Ministério da Saúde do Bras. 2017:1–7. Available from: http://portalarquivos.saude.gov.br/images/pdf/2017/junho/02/COES-FEBRE-AMARELA%2D%2D-INFORME-43%2D%2D-Atualiza%2D%2D%2D%2Do-em-31maio2017.pdf [cited 2019 Aug 1]

[5] MS-BR. Monitoramento do Período Sazonal da Febre Amarela Brasil – 2017/2018 - informe n^o^ 27 | 2017/2018 [Internet]. Ministério da Saúde do Bras. 2018:1–24. Available from: http://portalarquivos2.saude.gov.br/images/pdf/2018/outubro/08/Informe-FA.pdf [cited 2019 Jul 5]

[6] MS-BR. Monitoramento de Febre Amarela Brasil 2019 - informe n^o^ 18 | 9 de Junho 2019 [Internet]. Ministério da Saúde do Bras. 2019:1–8. Available from: https://portalarquivos2.saude.gov.br/images/pdf/2019/junho/13/Informe-de-Monitoramento-de-Febre-Amarela-Brasil%2D%2Dn-18.pdf [cited 2019 Aug 12]

[7] Hanley KA, Monath TP, Weaver SC, Rossi SL, Richman RL, Vasilakis N (2013). Fever versus fever: the role of host and vector susceptibility and interspecific competition in shaping the current and future distributions of the sylvatic cycles of dengue virus and yellow fever virus. Infect Genet Evol.

[8] Klitting R, Gould E, Paupy C, de Lamballerie X (2018). What Does the Future Hold for Yellow Fever Virus? (I). Genes (Basel).

[9] Moreno ES, Spinola R, Tengan CH, Brasil RA, Siciliano MM, Coimbra TLM (2013). Epizootias de febre amarela em primatas não humanos no estado de São Paulo, Brasil, 2008-2009. Rev Inst Med Trop Sao Paulo.

[10] Hudson NP, Philip CB (1929). Infective of blood during the course of experimental yellow fever. J Exp Med.

[11] Lindenbach BD, Murray CL, Thiel HJ, Rice CM, Knipe DM, Howley PM (2013). Flaviviridae. Fields Virol.

[12] Douam F, Ploss A. Yellow Fever Virus: Knowledge Gaps Impeding the Fight Against an Old Foe. Trends Microbiol. 2018;XX:1–16. 10.1016/j.tim.2018.05.012 PMC6340642 29933925

[13] Monath Thomas P., Vasconcelos Pedro F.C. (2015). Yellow fever. Journal of Clinical Virology.

[14] Espinoza Villar JC, Ronchail J, Guyot JL, Cochonneau G, Naziano F, Lavado W (2009). Spatio-temporal rainfall variability in the Amazon basin countries (Brazil, Peru, Bolivia, Colombia, and Ecuador). Int J Climatol.

[15] Roig C, Miret J, Rojas A, Guillén Y, Aria L, Mendoza L (2009). Estudio de Fiebre Amarilla en primates en áreas de brote de los departamentos de San Pedro y Central del Paraguay. Memorias Del Inst Investig en Ciencias la Salud.

[16] Bryant Juliet E., Holmes Edward C., Barrett Alan D. T. (2007). Out of Africa: A Molecular Perspective on the Introduction of Yellow Fever Virus into the Americas. PLoS Pathogens.

[17] Mir D, Delatorre E, Bonaldo M, Lourenço-De-Oliveira R, Vicente AC, Bello G (2017). Phylodynamics of yellow fever virus in the Americas: new insights into the origin of the 2017 Brazilian outbreak. Sci Rep.

[18] Vasconcelos PFC, Bryant JE, Travassos APA, Tesh RB, Rodrigues SG, Barrett ADT. Genetic divergence and dispersal of yellow fever virus. Emerg Infect Dis. 2004;10(9):1578. 10.3201/eid1009.040197 PMC3320275 15498159

[19] Bonaldo MC, Gómez MM, Dos Santos AAC, De Abreu FVS, Ferreira-de-Brito A, De Miranda RM (2017). Genome analysis of yellow fever virus of the ongoing outbreak in Brazil reveals polymorphisms. Mem Inst Oswaldo Cruz.

[20] Cunha MS, da Costa AC, de Azevedo Fernandes NCC, Guerra JM, dos Santos FCP, Nogueira JS (2019). Epizootics due to yellow fever virus in São Paulo state, Brazil: viral dissemination to new areas (2016–2017). Sci Rep.

[21] Faria NR, Kraemer MUG, Hill SC, De Jesus JG, Aguiar RS, Iani FCM, et al. Genomic and epidemiological monitoring of yellow fever virus transmission potential. Science. 2018;361:894–9. 10.1126/science.aat7115 PMC6874500 30139911

[22] Gómez MM, de Abreu FVS, Dos Santos AAC, de Mello IS, Santos MP, Ribeiro IP (2018). Genomic and structural features of the yellow fever virus from the 2016-2017 Brazilian outbreak. J Gen Virol.

[23] Quaresma JAS, Pagliari C, Medeiros DBA, Duarte MIS, Vasconcelos PFC (2013). Immunity and immune response, pathology and pathologic changes: progress and challenges in the immunopathology of yellow fever. Rev Med Virol.

[24] Tuboi Suely Hiromi, Costa Zouraide Guerra Antunes, da Costa Vasconcelos Pedro Fernando, Hatch Douglas (2007). Clinical and epidemiological characteristics of yellow fever in Brazil: analysis of reported cases 1998–2002. Transactions of the Royal Society of Tropical Medicine and Hygiene.

[25] Ho Y-L, Joelsons D, Leite GFC, Malbouisson LMS, Song ATW, Perondi B, et al. Severe yellow fever in Brazil: clinical characteristics and management. J Travel Med. 2019. 10.1093/jtm/taz040 31150098

[26] Ribeiro Ana Freitas, Cavalin Roberta Figueiredo, Abdul Hamid Suleiman Jamal Muhamad, Alves da Costa Jessica, Januaria de Vasconcelos Marileide, Sant’Ana Málaque Ceila Maria, Sztajnbok Jaques (2019). Yellow Fever: Factors Associated with Death in a Hospital of Reference in Infectious Diseases, São Paulo, Brazil, 2018. The American Journal of Tropical Medicine and Hygiene.

[27] Kallas Esper G, D'Elia Zanella Luiz Gonzaga F A B, Moreira Carlos Henrique V, Buccheri Renata, Diniz Gabriela B F, Castiñeiras Anna Carla P, Costa Priscilla R, Dias Juliana Z C, Marmorato Mariana P, Song Alice T W, Maestri Alvino, Borges Igor C, Joelsons Daniel, Cerqueira Natalia B, Santiago e Souza Nathália C, Morales Claro Ingra, Sabino Ester C, Levi José Eduardo, Avelino-Silva Vivian I, Ho Yeh-Li (2019). Predictors of mortality in patients with yellow fever: an observational cohort study. The Lancet Infectious Diseases.

[28] Casadio LVB, Salles APM, Malta FDM, Leite GF, Ho YL, Gomes-Gouvêa MS, et al. Lipase and factor V (but not viral load) are prognostic factors for the evolution of severe yellow fever cases. Mem Inst Oswaldo Cruz. 2019;114:e190033. 10.1590/0074-02760190033 PMC6528381 31116245

[29] Marinho Paula E.S., Alvarenga Pedro P.M., Crispim Ana P.C., Candiani Talitah M.S., Alvarenga Alice M., Bechler Isabela M., Alves Pedro A., Dornas Fabio P., de Oliveira Danilo B., Bentes Aline A., Christo Paulo P., Kroon Erna G. (2019). Wild-Type Yellow Fever Virus RNA in Cerebrospinal Fluid of Child. Emerging Infectious Diseases.

[30] Barbosa Carla M., Di Paola Nicholas, Cunha Marielton P., Rodrigues-Jesus Mônica J., Araujo Danielle B., Silveira Vanessa B., Leal Fabyano B., Mesquita Flávio S., Botosso Viviane F., Zanotto Paolo M.A., Durigon Edison L., Silva Marcos V., Oliveira Danielle B.L. (2018). Yellow Fever Virus DNA in Urine and Semen of Convalescent Patient, Brazil. Emerging Infectious Diseases.

[31] Denis B, Chirio D, Ponscarme D, Brichler S, De Verdière NC, Simon F, et al. Hepatitis rebound after infection with yellow fever virus. Emerg Infect Dis. 2019;25(6):1248. 10.3201/eid2506.190069 PMC6537733 30870138

[32] MS-BR. GUIA DE VIGILÂNCIA EM SAÚDE [Internet]. Ministério da Saúde do Bras. 2019 p. 1–706. Available from: http://bvsms.saude.gov.br/bvs/publicacoes/guia_vigilancia_saude_3ed.pdf[cited 2019 Jul 13]

[33] MS-BR. Guia de vigilância de epizootias em primatas não humanos e entomologia aplicada à vigilância da febre amarela [Internet]. 2nd ed. Ministério da Saúde do Bras. 2017. Available from: http://portalarquivos.saude.gov.br/images/pdf/2017/marco/24/Guia_Epizootias_Febre_Amarela_2a_ed_atualizada_2017.pdf [cited 2019 Jul 2]

[34] Favoretto S, Araujo D, Oliveira D, Duarte N, Mesquita F, Zanotto P, et al. First detection of Zika virus in neotropical primates in Brazil: a possible new reservoir. bioRxiv. 2016.

[35] Terzian ACB, Zini N, Sacchetto L, Rocha RF, Parra MCP, Del Sarto JL, et al. Evidence of natural Zika virus infection in neotropical non-human primates in Brazil. Sci Rep. 2018;8(1):16034. 10.1038/s41598-018-34423-6 PMC6207778 30375482

[36] PAHO-WHO. Laboratory Diagnosis of Yellow Fever Virus infection [Internet]. Pan Am Heal Organ. World Heal Organ. 2018:1–8. Available from: https://www.paho.org/hq/index.php?option=com_docman&view=download&category_slug=guidelines-5053&alias=46877-laboratory-diagnosis-of-yellow-fever-virus-infection&Itemid=270&lang=en [cited 2019 Jun 5].

[37] PAHO. Control of Yellow Fever [Internet]. Pan Am Heal. Organ. 2005:1–66. Available from: http://www.paho.org/immunization/toolkit/resources/paho-publication/field-guides/Control-of-Yellow-Fever.pdf?ua=1 [cited 2019 Jun 2].

[38] Monath TP (2005). Yellow fever vaccine. Expert Rev Vaccines.

[39] WHO. Weekly epidemiological record Relevé épidémiologique hebdomadaire [Internet]. World Heal Organ. 2013:269–84 Available from: https://www.who.int/wer/2013/wer8827.pdf?ua=1&ua=1%3E.[cited 2019 Jul 2].

[40] MS-BR. Orientações e indicação de dose única da vacina febre amarela - Nota Informativa N° 94 [Internet]. Ministério da Saúde do Bras. 2017 .Available from: http://www.saude.mg.gov.br/images/documentos/Nota Informativa dose única FA.pdf.[cited 2019 Jul 18].

[41] Collaborative group for studies on yellow fever vaccines. Duration of post-vaccination immunity against yellow fever in adults. Vaccine. 2014;32(39):4977–84. 10.1016/j.vaccine.2014.07.021 25090646

[42] Campi-Azevedo AC, Costa-Pereira C, Antonelli LR, Fonseca CT, Teixeira-Carvalho A, Villela-Rezende G (2016). Booster dose after 10 years is recommended following 17DD-YF primary vaccination. Hum Vaccines Immunother.

[43] Costa-Pereira Christiane, Campi-Azevedo Ana Carolina, Coelho-dos-Reis Jordana Grazziela, Peruhype-Magalhães Vanessa, Araújo Márcio Sobreira Silva, do Vale Antonelli Lis Ribeiro, Fonseca Cristina Toscano, Lemos Jandira Aparecida, Malaquias Luiz Cosme Cote, de Souza Gomes Matheus, Rodrigues Amaral Laurence, Rios Maria, Chancey Caren, Persi Harold Richard, Pereira Jorge Marcelo, de Sousa Maia Maria de Lourdes, Freire Marcos da Silva, Martins Reinaldo de Menezes, Homma Akira, Simões Marisol, Yamamura Anna Yoshida, Farias Roberto Henrique Guedes, Romano Alessandro Pecego Martins, Domingues Carla Magda, Tauil Pedro Luiz, Vasconcelos Pedro Fernando Costa, Caldas Iramaya Rodrigues, Camacho Luiz Antônio, Teixeira-Carvalho Andrea, Martins-Filho Olindo Assis (2018). Multi-parameter approach to evaluate the timing of memory status after 17DD-YF primary vaccination. PLOS Neglected Tropical Diseases.

[44] WHO. Detection and investigation of serious adverse events following yellow fever vaccination - Guidance from an informal consultation of experts [Internet]. World Heal Organ. 2008:1–62 Available from: https://apps.who.int/iris/bitstream/handle/10665/70251/WHO_HSE_GAR_ERI_2010.2_eng.pdf?sequence=1%3E. [cited 2019 Jul 18].

[45] MS-BR. Manual de vigilância epidemiológica de eventos adversos pós-vacinação [Internet]. Ministério da Saúde do Bras. 2014:1–254. Available from: http://bvsms.saude.gov.br/bvs/publicacoes/manual_vigilancia_epidemiologica_eventos_adversos_pos_vacinacao.pdf [cited 2019 Jul 17]

[46] SES-MG. Eventos adversos pós-vacinação associados à vacina febre amarela (EAPV-VFA) Minas Gerais, 2016 a 2018 [Internet]. Secr. Estado Saúde Minas Gerais. 2018: 1–13. Available from: http://www.saude.mg.gov.br/images/noticias_e_eventos/000_2018/BoletinsEpidemiologicos/Boletim_epidemiol%F3gico_EAPV_finalv3.pdf [cited 2019 Jul 2]

[47] Selemane Ismael (2019). Epidemiological monitoring of the last outbreak of yellow fever in Brazil – An outlook from Portugal. Travel Medicine and Infectious Disease.

[48] Prata A. Yellow Fever (1976). 2000(95):183–7. [internet] Available from: https://felakuti.bandcamp.com/album/yellow-fever-1976. 10.1590/s0074-02762000000700031 11142712

[49] Soper FL (1938). Yellow Fever in the Americas, 1938–1942. Febre Amarela Panam.

[50] Franco O. História da Febre Amarela do Brasil [Internet]. Ministério da Saúde Dep. Nac. Endem. Rurais. 1969:1–212. Available from: http://bvsms.saude.gov.br/bvs/publicacoes/0110historia_febre.pdf [cited 2019 Jul 18]

[51] SOPER F. L., PENNA H., CARDOSO E., SERAFIM J., FROBISHER M., PINHEIRO J. (1933). YELLOW FEVER WITHOUT AËDES AEGYPTI. STUDY OF A RURAL EPIDEMIC IN THE VALLE DO CHANAAN, ESPIRITO SANTO, BRAZIL, 1932*. American Journal of Epidemiology.

[52] MS-BR. Guia de Vigilância em Saúde [Internet]. Ministério da Saúde do Bras. - Secr. Vigilância em Saúde. 2017:1–706. Available from: http://portalarquivos.saude.gov.br/images/pdf/2017/outubro/06/Volume-Unico-2017.pdf [cited 2019 Jul 18]

[53] Delatorre E, de Abreu FVS, Ribeiro IP, Gómez MM, dos Santos AAC, Ferreira-de-Brito A (2019). Distinct YFV lineages co-circulated in the Central-Western and southeastern Brazilian regions from 2015 to 2018. Front Microbiol.

[54] Rezende Izabela Maurício de, Sacchetto Lívia, Munhoz de Mello Érica, Alves Pedro Augusto, Iani Felipe Campos de Melo, Adelino Talita Émile Ribeiro, Duarte Myrian Morato, Cury Ana Luísa Furtado, Bernardes André Felipe Leal, Santos Tayrine Araújo, Pereira Leonardo Soares, Dutra Maria Rita Teixeira, Ramalho Dario Brock, de Thoisy Benoit, Kroon Erna Geessien, Trindade Giliane de Souza, Drumond Betânia Paiva (2018). Persistence of Yellow fever virus outside the Amazon Basin, causing epidemics in Southeast Brazil, from 2016 to 2018. PLOS Neglected Tropical Diseases.

[55] SES-MG. Atualização: Situação epidemiológica da febre amarela silvestre em Minas Gerais, 2017 [Internet]. Secr. Estado Saúde Minas Gerais. 2017:1–25. Available from: http://www.saude.mg.gov.br/images/icones/Atualizao FA - 29 de Junho 2017.pdf. [cited 2019 Jun 8]

[56] SES-MG. Febre Amarela em Minas Gerais - Boletim epidemiológico – 30/01/2018 [Internet]. Secr. Estado Saúde Minas Gerais. 2018:1–9. Available from: http://www.saude.mg.gov.br/images/noticias_e_eventos/000_2018/Boletim_Febre_Amarela__30.01.2018.pdf. [cited 2019 Aug 13]

[57] de Abreu FVS, Delatorre E, Dos Santos AAC, Ferreira-de-Brito A, de Castro MG, Ribeiro IP (2019). Combination of surveillance tools reveals that yellow fever virus can remain in the same Atlantic Forest area at least for three transmission seasons. Mem Inst Oswaldo Cruz.

[58] G1BR. Macacos são vítimas da falta de informação sobre febre amarela [Internet]. Globo. 2018. Available from: http://g1.globo.com/jornal-nacional/noticia/2018/01/macacos-sao-vitimas-da-falta-de-informacao-sobre-febre-amarela.html [cited 2019 Jun 2]

[59] QGIS Development Team. Welcome to the QGIS project! Qgis. 2016.

[60] SESAB-BA. Boletim Epidemiológico da Febre Amarela, N^o^ 2 [Internet]. Secretária da Saúde da Bahia - Dir. Vigilância Epidemiológica—Divep. 2018:1–4. Available from: http://www.saude.ba.gov.br/wp-content/uploads/2017/11/2018-Boletim-epidemiologico-da-Febre-Amarela-n-02.pdf [cited 2019 Jun 27].

[61] SES-SP. Boletim Epidemiológio Febre Amarela 28/12/2018 [Internet]. Secr. Estado da Saúde - Gov. São Paulo. 2018:1–9. Available from: http://www.saude.sp.gov.br/resources/cve-centro-de-vigilancia-epidemiologica/areas-de-vigilancia/doencas-de-transmissao-por-vetores-e-zoonoses/doc/famarela/fa18_boletim_epid_2812.pdf [cited 2019 Jun 27]

[62] SES-SP. Boletim Epidemiológio Febre Amarela – 21/01/2019 [Internet]. Secr. Estado da Saúde - Gov. São Paulo. 2019:1–5. Available from: http://www.saude.sp.gov.br/resources/cve-centro-de-vigilancia-epidemiologica/areas-de-vigilancia/doencas-de-transmissao-por-vetores-e-zoonoses/doc/famarela/2019/fa19_boletim_epid_210119.pdf [cited 2019 Jun 27]

[63] SES-SP. Boletim Epidemiológio Febre Amarela – 03/06/2019 [Internet]. Secr. Estado da Saúde - Gov. São Paulo. 2019:1–6. Available from: http://www.saude.sp.gov.br/resources/cve-centro-de-vigilancia-epidemiologica/areas-de-vigilancia/doencas-de-transmissao-por-vetores-e-zoonoses/doc/famarela/2019/fa19_boletim_epid_0306.pdf

[64] SESAB-BA. Boletim Epidemiológico de Febre Amarela, Bahia, 2019, N^o^1 [Internet]. Secr. da Saúde do Estado da Bahia. 2019:1–3. Available from: http://www.saude.ba.gov.br/wp-content/uploads/2017/11/2019-Boletim-epidemiol%F3gico-da-Febre-Amarela-n.01.pdf [cited 2019 Jun 27]

[65] COES-PR. Boletim EpidemiológicoN^o^ 015–30/05/2019 Período sazonal: Julho/2018 a Junho/2019 [Internet]. Cent. OPERAÇÕES EMERGÊNCIAS EM SAÚDE PÚBLICA - Gov. do Estado do Paraná. 2019:1–6. Available from: http://www.saude.pr.gov.br/arquivos/File/BoletimEpidemiologico_15FA.pdf [cited 2019 Jun 27]

[66] COES-PR. Boletim Epidemiológico N^o^ 017–04/07/2019 Período sazonal: Julho/2018 a Junho/2019 [Internet]. Cent. OPERAÇÕES EMERGÊNCIAS EM SAÚDE PÚBLICA - Gov. do Estado do Paraná. 2019:1–5. Available from: http://www.saude.pr.gov.br/arquivos/File/BoletimEpidemiologico_17FA.pdf [cited 2019 Jun 27]

[67] SES-PR. Boletim Epidemiológio Febre Amarela N^o^ 08/2018–03 de Julho de 2018 [Internet]. Secr. Estado da Saúde do Paraná. 2018:1–9. Available from: http://www.saude.pr.gov.br/arquivos/File/BoletimFA07.pdf [cited 2019 Jun 27]

[68] SES-SC. Boletim Epidemiológico da Febre Amarela n° 10/2019–29 de junho de 2019 Período de monitoramento (julho/2018 a junho/2019) [Internet]. Secr. Estado da Saúde - Gov. St. Catarina. 2018 [cited 2019 Jun 27]:1–7. Available from: Boletim Epidemiológico da Febre Amarela n° 10/2019–29 de junho de 2019%0APeríodo de monitoramento (julho/2018 a junho/2019).

[69] SES-SC. Boletim Epidemiológico Febre Amarela n° 18/2018–18 de dezembro de 2018 [Internet]. Secr. Estado da Saúde - Gov. St. Catarina. 2018:1–5. Available from: http://www.dive.sc.gov.br/conteudos/boletim2018/boletimFebreAmarela18/Bolet18Febre Amarela.pdf.[cited 2019 Jun 27]

[70] SES-RJ. Informe Epidemiológico – Febre Amarela [Internet]. Secr. da Saúde do Rio Janeiro. 2018. Available from: http://www.febreamarelarj.com.br/comum/code/MostrarArquivo.php? C=NjM%2C.[cited 2019 Jun 27]

[71] SES-SP. Boletim Epidemiológio Febre Amarela 08/01/2018 [Internet]. Secr. Estado da Saúde - Gov. São Paulo. 2018:1–8. Available from: http://www.saude.sp.gov.br/resources/cve-centro-de-vigilancia-epidemiologica/areas-de-vigilancia/doencas-de-transmissao-por-vetores-e-zoonoses/doc/famarela/fa18_boletim_epid_0801.pdf [cited 2019 Jun 27]

[72] SES-MG. Febre Amarela Silvestre em Minas Gerais, Boletim epidemiológico 19/02/2019 [Internet]. Secr. Estado Saúde Minas Gerais. 2019:1–8. Available from: http://www.saude.mg.gov.br/images/noticias_e_eventos/000_2019/jane_fev_mar/Febre_Amarela/Boletim_atualiza%E7%E3o_FA_12-02-2019.pdf [cited 2019 Aug 13]

[73] SES-MG. Boletim epidemiológico – 20/06/2018 Febre Amarela Silvestre em Minas Gerais [Internet]. Secr. Estado Saúde Minas Gerais. 2018. Available from: http://www.saude.mg.gov.br/images/documentos/Boletim _Febre Amarela_21.06.2018_atualizada.pdf. [cited 2019 Jun 2]

[74] Germain M., Mouchet J., Cordellier R., Chippaux A., Cornet M., Herve J.P., Sureau P., Fabre J., Robin Y. (1978). Épidémiologie de la fièvre jaune en Afrique. Médecine et Maladies Infectieuses.

[75] Abreu FVS, Ribeiro IP, Ferreira-de-Brito A, dos SAAC, de Miranda RM, de S BI, et al. *Haemagogus leucocelaenus* and *Haemagogus janthinomys* are the primary vectors in the major yellow fever outbreak in Brazil, 2016–2018. Emerg Microbes Infect. 2019;8:218–31. 10.1080/22221751.2019.1568180 PMC6455131 30866775

[76] Cardoso JC, de Almeida MAB, dos Santos E, da Fonseca DF, Sallum MAM, Noll CA, et al. Yellow fever virus in *Haemagogus leucocelaenus* and *Aedes serratus* mosquitoes, southern Brazil, 2008. Emerg Infect Dis. 2010;16:1918–24. 10.3201/eid1612.100608 PMC3294583 21122222

[77] Couto-Lima D, Madec Y, Bersot MI, Campos SS, de Albuquerque MM, Dos Santos FB, et al. Potential risk of re-emergence of urban transmission of Yellow Fever virus in Brazil facilitated by competent *Aedes* populations. Sci Rep. 2017;7:1–12. 10.1038/s41598-017-05186-3 PMC5501812 28687779

[78] Monath TP, Monath TP (1989). Yellow fever. Arboviruses Ecol Epidemiol.

[79] Whitman Loring, Antunes P. C. A. (1938). The Transmission of Two Strains of Jungle Yellow Fever Virus by Aëdes Aegypti 1. The American Journal of Tropical Medicine and Hygiene.

[80] Alencar Jeronimo, Lorosa Elias Seixas, Dégallier Nicolas, Serra-Freire Nicolau Maués, Pacheco Juliana Barreto, Guimarães Anthony Érico (2005). Feeding Patterns ofHaemagogus janthinomys(Diptera: Culicidae) in Different Regions of Brazil. Journal of Medical Entomology.

[81] WHO. Geographical distribution of arthropod-borne diseases and their principal vectors [Internet]. World Heal. Organ Vector Biol. Control Div. 1989:1–134. Available from: https://apps.who.int/iris/bitstream/handle/10665/60575/WHO_VBC_89.967.pdf?sequence=1&isAllowed=y]. [cited 2019 Jun 20]

[82] Degallier N, da Rosa APA T, Herve JP (1992). A comparative study of yellow fever in Africa and South America. Cienc Cult.

[83] Alencar J, de Mello CF, Barbosa LS, Gil-Santana HR, de A MD, Marcondes CB (2016). Diversity of yellow fever mosquito vectors in the Atlantic forest of Rio de Janeiro, Brazil. Rev Soc Bras Med Trop.

[84] Forattini OP. Culicidologia Médica - Identificação, biologia e epidemiologia. Culicidologia Médica. 2002.

[85] Marcondes C, Alencar J. Revisão de mosquitos *Haemagogus Williston* (Diptera: Culicidae) do Brasil. Rev Biomed. 2010.

[86] WHO. Risk assessment on yellow fever virus circulation in endemic countries - WHO [Internet]. World Heal. Organ. 2014:1–40. Available from: https://apps.who.int/iris/bitstream/handle/10665/112751/WHO_HSE_PED_CED_2014.2_eng.pdf?sequence=1 [cited 2019 Jun 5]

[87] Hervé J-P. Travassos da Rosa APA. Ecologia da febre amarela no Brasil. Rev da Fund SESP, Rio Janeiro. 1983;28(1):11–9.

[88] Gomes AC, Torres MAN T, Ferri L, da Costas FR, da Silva AM. Encontro de *Haemagogus* (Conopostegus) *leucocelaenus* (Diptera: Culicidae), no Município de Porto Alegre, Estado do Rio Grande do Sul. Rev Soc Bras Med Trop. 2007;40:487–8. 10.1590/s0037-86822007000400025 17876479

[89] Possas C, Lourenço-de-Oliveira R, Tauil PL, Pinheiro FP, Pissinatti A, da Cunha RV, et al. Yellow fever outbreak in Brazil: the puzzle of rapid viral spread and challenges for immunisation. Mem Inst Oswaldo Cruz. 2018;113:e180278. 10.1590/0074-02760180278 PMC6135548 30427974

[90] Causey OR, Kumm HW, Laemmert HW (1950). Dispersion of forest mosquitoes in Brazil; further studies. Am J Trop Med Hyg..

[91] Mucci Luis Filipe, Medeiros-Sousa Antônio Ralph, Ceretti-Júnior Walter, Fernandes Aristides, Camargo Amanda Alves, Evangelista Eduardo, de Oliveira Christe Rafael, Montes Joyce, Teixeira Renildo Souza, Marrelli Mauro Toledo (2016). Haemagogus leucocelaenusand Other Mosquitoes Potentially Associated With Sylvatic Yellow Fever In Cantareira State Park In the São Paulo Metropolitan Area, Brazil. Journal of the American Mosquito Control Association.

[92] Pinheiro GG, Rocha MN, de Oliveira MA, Moreira LA, Filho JDA (2019). Detection of yellow fever virus in sylvatic mosquitoes during disease outbreaks of 2017-2018 in Minas Gerais state, Brazil. Insects.

[93] Pereira dos Santos Taissa, Roiz David, Santos de Abreu Filipe Vieira, Luz Sergio Luiz Bessa, Santalucia Marcelo, Jiolle Davy, Santos Neves Maycon Sebastiao Alberto, Simard Frédéric, Lourenço-de-Oliveira Ricardo, Paupy Christophe (2018). Potential of Aedes albopictus as a bridge vector for enzootic pathogens at the urban-forest interface in Brazil. Emerging Microbes & Infections.

[94] Miller Barry R., Ballinger Mary E. (1988). Aedes albopictus mosquitoes introduced into Brazil: vector competence for yellow fever and dengue viruses. Transactions of the Royal Society of Tropical Medicine and Hygiene.

[95] Massad Eduardo, Coutinho Francisco Antonio Bezerra, Burattini Marcelo Nascimento, Lopez Luiz Fernandes (2001). The risk of yellow fever in a dengue-infested area. Transactions of the Royal Society of Tropical Medicine and Hygiene.

[96] Johnson Barbara W., Chambers Trudy V., Crabtree Mary B., Filippis Ana M.B., Vilarinhos Paulo T.R., Resende Marcelo C., Macoris Maria de Lourdes G., Miller Barry R. (2002). Vector competence of Brazilian Aedes aegypti and Ae. albopictus for a Brazilian yellow fever virus isolate. Transactions of the Royal Society of Tropical Medicine and Hygiene.

[97] IEC. Instituto Evandro Chagas detecta vírus da Febre Amarela em mosquito *Aedes albopictus* no Brasil [Internet]. Inst. Evandro Chagas. 2018 . Available from: https://www.iec.gov.br/descoberta/

[98] Lourenço-de-Oliveira Ricardo, Vazeille Marie, Filippis Ana Maria Bispo de, Failloux Anna-Bella (2002). Oral Susceptibility to Yellow Fever Virus of Aedes aegypti from Brazil. Memórias do Instituto Oswaldo Cruz.

[99] Lourenço-de-Oliveira R, Vazeille M, de Filippis A.M.B, Failloux A.B (2004). Aedes aegypti in Brazil: genetically differentiated populations with high susceptibility to dengue and yellow fever viruses. Transactions of the Royal Society of Tropical Medicine and Hygiene.

[100] Mittermeier, RA; Rylands, AB; Wilson, DE (Chief Eds.): Handbook of the Mammals of the World, vol. 3. Primates, Lynx Edicions, Barcelona. 2013:951.

[101] Dunn J, Cristóbal-Azkarate J. New World Monkeys [Internet]. Nat. Educ. 2016. Available from: https://www.nature.com/scitable/knowledge/library/new-world-monkeys-148121150/ [cited 2019 Jun 1].

[102] Paglia AP, Fonseca GAB, Rylands AB, Herrmann G, Aguiar LMS, Chiarello AG, et al. Lista anotada dos mamíferos do Brasil Occas. Pap. Conserv. Biol. 2nd. Arlington, VA: Conservation International; 2012.

[103] Bicca-Marques Júlio César, de Freitas David Santos (2010). The Role of Monkeys, Mosquitoes, and Humans in the Occurrence of a Yellow Fever Outbreak in a Fragmented Landscape in South Brazil: Protecting Howler Monkeys is a Matter of Public Health. Tropical Conservation Science.

[104] Duarte Marina H.L., Goulart Vinícius D.L.R., Young Robert J. (2012). Designing laboratory marmoset housing: What can we learn from urban marmosets?. Applied Animal Behaviour Science.

[105] Teixeira B, Hirsch A, Goulart VDLR, Passos L, Teixeira CP, James P, et al. Good neighbours: distribution of black-tufted marmoset (*Callithrix penicillata*) in an urban environment. Wildl Res. 2015;42:579–89.

[106] Daviews C. R., Ayres J. M., Dye C., Deane L. M. (1991). Malaria Infection Rate of Amazonian Primates Increases with Body Weight and Group Size. Functional Ecology.

[107] Neville MK, Glander KE, Braza F, Rylands AB. The howling monkeys, genus Alouatta. In: Mittermeier, R. A., Rylands, A. B., Coimbra-Filho, A. F. and Fonseca GAB, editor. Ecol Behav Neotrop Primates, vol 2. Washington, DC: World Wildlife Fundation; 1988:348–453.

[108] de Thoisy B, Richard-Hansen C. Diet and social behaviour changes in a red howler monkey (*Alouatta seniculus*) troop in a highly degraded rain forest. Folia Primatol. 1997;68:357–61.

[109] Woodall J. P., Dykes J. R. W., Williams M. C. (1968). The reaction of a species of colobus monkey to inoculation with yellow fever virus. Annals of Tropical Medicine & Parasitology.

[110] Taufflieb R, Robin Y, Cornet M. Le virus amaril et la faune sauvage en Afrique. Cahiers. ORSTOM Série Entomol Médicale Parasitol. 1971;9:351–71.

[111] Taylor RM, Haseeb MA, Work TH (1955). A regional reconnaissance on yellow fever in the Sudan. With special reference to primate hosts. Bull World Health Organ.

[112] Davis NC (1930). Susceptibility of capuchin (Cebus) monkeys to yellow fever virus. Am J Epidemiol.

[113] Davis NC (1930). The susceptibility of marmosets to yellow fever virus. J Exp Med.

[114] Davis NC. The transmission of yellow fever: experiments with the “woolly monkeys” (*Lagothrix Lagotricha* Humboldt), the “spider monkey” (*Ateleus Ater* F. Cuvier), and the “squirrel monkey” (*Saimiri Scireus *Linnaeus). J Exp Med. 1931;51:703–21. 10.1084/jem.51.5.703 PMC2131789 19869721

[115] Holzmann I, Agostini I, Areta JI, Ferreyra H, Beldomenico P, di Bitetti MS. Impact of yellow fever outbreaks on two howler monkey species (*Alouatta Guariba* clamitans and *A. caraya*) in Misiones, Argentina. Am J Primatol. 2010;72:475–80. 10.1002/ajp.20796 20095025

[116] Ott-Joslin JE. Viral diseases in nonhuman primates. In: M F, editor. Zoo wild Anim Med. Philadelphia: W.B. Saunders; 1986:674–697.

[117] Burke A. W. (1937). An Epidemic of Jungle Yellow Fever on the Planalto of Matto Grosso, Brazil 1. The American Journal of Tropical Medicine and Hygiene.

[118] Laemmert H. W., Causey O. R., Hughes T. P. (1949). The Invasion of Small Forests by Yellow Fever Virus as Indicated by Immunity in Cebus Monkeys 1. The American Journal of Tropical Medicine and Hygiene.

[119] Laemmert Jr HW, Ferreira LC, Taylor RM. An epidemiological Study of jungle yellow fever in a endemic area in Brazil. Part II – Investigations of vertebrate hosts and arthropod vectors. Am Soc Trop Med Hyg. 1946;s1–26:23–69. 20279501

[120] Kumm HW, Laemmert Jr. HW. The Geographical Distribution of Immunity to Yellow Fever among the Primates of Brazil. Am Soc Trop Med Hyg. 1950;s1–30:733–748. 10.4269/ajtmh.1950.s1-30.733 14771397

[121] Strier Karen B., Tabacow Fernanda P., de Possamai Carla B., Ferreira Anderson I. G., Nery Marcello S., de Melo Fabiano R., Mendes Sérgio L. (2018). Status of the northern muriqui (Brachyteles hypoxanthus) in the time of yellow fever. Primates.

[122] Laemmert Hugo W., Kumm Henry W. (1950). The Susceptibility of Howler Monkeys to Yellow Fever Virus 1. The American Journal of Tropical Medicine and Hygiene.

[123] Galindo P. Monkeys and yellow fever. In: Nonhum Primates Med Res. 3rd ed: Academic Press, Inc. 1973.

[124] De Thoisy B, Vogel I, Reynes JM, Pouliquen JF, Carme B, Kazanji M (2001). Health evaluation of translocated free-ranging primates in French Guiana. Am J Primatol.

[125] Pinheiro FP, da Rosa APA T, da Rosa MAP T (1981). An Epidemic of Yellow Fever in Central Brazil, 1972–1973. II. Ecological Studies. Am Soc Trop Med Hyg.

[126] Agostini Ilaria, Holzmann Ingrid, Di Bitetti Mario S. (2008). Infant hybrids in a newly formed mixed-species group of howler monkeys (Alouatta guariba clamitans and Alouatta caraya) in northeastern Argentina. Primates.

[127] Vargas-Mendez O, Elton NW (1953). Naturally acquired yellow fever in wild monkeys of Costa Rica^1^. Am J Trop Med Hyg..

[128] Collias N, Southwick C (1952). A field study of population density and social organization in howling monkeys. Am Philos Soc.

[129] Auguste AJ, Lemey P, Bergren NA, Giambalvo D, Moncada M, Morón D (2015). Enzootic transmission of yellow fever virus. Venezuela Emerg Infect Dis.

[130] Rifakis PM, Benitez JA, De-La-Paz-Pineda J, Rodriguez-Morales AJ (2006). Epizootics of yellow fever in Venezuela (2004-2005): an emerging zoonotic disease. Ann N Y Acad Sci.

[131] De Almeida MAB, Dos Santos E, Da Cruz CJ, Da Fonseca DF, Noll CA, Silveira VR (2012). Yellow fever outbreak affecting Alouatta populations in southern Brazil (Rio Grande do Sul state), 2008-2009. Am J Primatol.

[132] Sallis Eliza Simone Viégas, de Barros Vera Lúcia Reis Souza, Garmatz Shana Letícia, Fighera Rafael Almeida, Graça Dominguita Lühers (2003). A Case of Yellow Fever in a Brown Howler (Alouatta Fusca) in Southern Brazil. Journal of Veterinary Diagnostic Investigation.

[133] Laemmert Hugo W., de Castro Ferreira Leoberto (1945). The Isolation of Yellow Fever Virus from Wild-Caught Marmosets 1. The American Journal of Tropical Medicine and Hygiene.

[134] Bates Brian C. (1970). Territorial behavior in primates: A review of recent field studies. Primates.

[135] Davis Nelson C., Shannon Raymond C. (1929). STUDIES ON SOUTH AMERICAN YELLOW FEVER. The Journal of Experimental Medicine.

[136] de Abreu FVS, dos Santos E, Gomes MQ, Vargas WP, Oliveira Passos PH, Nunes e Silva C, et al. Capture of *Alouatta guariba clamitans* for the surveillance of sylvatic yellow fever and zoonotic malaria: Which is the best strategy in the tropical Atlantic Forest? Am J Primatol. 2019: e23000. 10.1002/ajp.23000 31192493

[137] Figueiredo PO, Silva ATS, Oliveira JS, Marinho PE, Rocha FT, Domingos GP (2018). Detection and molecular characterization of yellow fever virus, 2017. Brazil Ecohealth.

[138] Fernandes NCC, Cunha MS, Guerra JM, Réssio RA, Cirqueira CS, S. DI (2017). Outbreak of yellow fever among nonhuman primates, Espirito Santo, Brazil, 2017. Emerg Infect Dis.

[139] Leal Silvana Gomes, Romano Alessandro Pecego Martins, Monteiro Rafael Veríssimo, Melo Cristiano Barros de, Vasconcelos Pedro Fernando da Costa, Castro Márcio Botelho de (2016). Frequency of histopathological changes in Howler monkeys ( Alouatta sp.) naturally infected with yellow fever virus in Brazil. Revista da Sociedade Brasileira de Medicina Tropical.

[140] STOKES ADRIAN, BAUER J. H., HUDSON N. PAUL (1928). THE TRANSMISSION OF YELLOW FEVER TO MACACUS RHESUS. Journal of the American Medical Association.

[141] Bearcroft WGC (1957). The histopathology of the liver of yellow fever infected rhesus monkeys. J Pathol.

[142] Engelmann Flora, Josset Laurence, Girke Thomas, Park Byung, Barron Alex, Dewane Jesse, Hammarlund Erika, Lewis Anne, Axthelm Michael K., Slifka Mark K., Messaoudi Ilhem (2014). Pathophysiologic and Transcriptomic Analyses of Viscerotropic Yellow Fever in a Rhesus Macaque Model. PLoS Neglected Tropical Diseases.

[143] Monath TP, Brinker KR, Chandler FW, Kemp GE, Cropp CB (1981). Pathophysiologic Correlations in a Rhesus Monkey Model of Yellow Fever With Special Observations on the Acute Necrosis of B Cell Areas of Lymphoid Tissues. Am Soc Trop Med Hyg.

